# Explainable multimodal fusion model integrating clinical and quantitative CT features for preoperative prediction of high-grade histologic patterns in invasive lung adenocarcinoma

**DOI:** 10.3389/fonc.2026.1910400

**Published:** 2026-08-17

**Authors:** Yiwen An, Beibei Hou, Zhizhen Ran, Peng Chen, Juxian Li, Zhan Yin, Jinling Zhang

**Affiliations:** 1 Department of Computed Tomography, The Second Affiliated Hospital of Harbin Medical University, Harbin Medical University, Harbin, Heilongjiang, China; 2 Department of Radiology, The Fourth Hospital of Harbin, Harbin, Heilongjiang, China; 3 Department of Radiology, The First Affiliated Hospital of Henan Medical University, Henan Medical University, Xinxiang, Henan, China

**Keywords:** 3D fractal dimension, high-grade patterns, intratumoral heterogeneity, invasive lung adenocarcinoma, shapley additive explanations

## Abstract

**Introduction:**

Accurate preoperative prediction of high-grade patterns in invasive lung adenocarcinoma is critical for effective treatment planning. This study aimed to construct an interpretable multimodal fusion model to predict high-grade pattern status by integrating clinical information and quantitative CT features, including radiomics, intratumoral heterogeneity, and three-dimensional fractal features.

**Methods:**

In this retrospective study, 743 patients with pathologically confirmed invasive lung adenocarcinoma from two centers were classified into positive and negative groups for high-grade patterns. Patients from the primary center (n=650) were divided into a training cohort (n=455) and an internal validation cohort (n=195), while the second center provided an external validation cohort (n=93). After extracting relevant clinical and imaging features, the optimal predictive model was identified from 80 combinations of various feature selection methods and machine learning algorithms.

**Results:**

The resulting multimodal fusion model demonstrated robust predictive performance, achieving an area under the curve of 0.848 (sensitivity 84.7%, specificity 71.1%) in the internal validation cohort and maintaining an area under the curve of 0.815 (sensitivity 85.1%, specificity 73.9%) in the external cohort. This integrated approach outperformed individual clinical or radiomics models, as well as standard classifiers such as XGBoost, logistic regression, and multilayer perceptrons. Decision curve analysis further demonstrated favorable potential clinical utility, with the combined model generally providing a higher net clinical benefit than the treat-all and treat-none strategies across the threshold probability range examined.

**Discussion:**

By employing Shapley Additive Explanations to ensure interpretability, this comprehensive predictive tool offers reliable support for preoperative risk stratification and facilitates more precise, individualized clinical decision-making.

## 1 Introduction

Lung cancer is the leading cause of cancer-related death worldwide (1). Non-small cell lung cancer accounts for approximately 80% of primary lung cancers, with lung adenocarcinoma (LUAD) being the most common histopathological subtype (2, 3). According to the 2021 World Health Organization Classification of Thoracic Tumors, LUAD is classified into adenocarcinoma *in situ*, minimally invasive adenocarcinoma and invasive adenocarcinoma (IAC). Within IAC, non-mucinous adenocarcinoma is predominant, exhibiting a variety of histological growth patterns such as lepidic, acinar, papillary, micropapillary, solid, and complex glandular patterns (4, 5). IAC is characterized by marked histological heterogeneity and is commonly composed of multiple histological subtypes rather than a single pattern (6). This pronounced spatial heterogeneity leads to substantial phenotypic and morphological variation across tumor subregions, posing challenges for accurate histological grading and prognostic assessment of LUAD.

The International Association for the Study of Lung Cancer Pathology Committee has introduced an innovative grading system that identifies high-grade patterns (HGP) as a central prognostic factor. In this system, tumors with ≥20% HGP are classified as Grade 3, indicating the most unfavorable prognosis (7). For small peripheral lesions, sublobar resections, including wedge resection and segmentectomy, are increasingly regarded as viable alternatives to standard lobectomy (8, 9). Furthermore, HGP are closely associated with aggressive tumor biological behavior, including lymphovascular invasion, spread through air spaces, and increased risk of dissemination (5, 10), suggesting that limited resection may be oncologically inadequate for tumors harboring HGP. Consequently, anatomical lobectomy accompanied by systematic lymph node dissection remains the standard surgical approach for patients exhibiting such aggressive histopathological features (11, 12). Due to inherent sampling bias, conventional preoperative core needle biopsy may be insufficient to fully capture intratumoral heterogeneity, thereby limiting diagnostic accuracy (13). Therefore, a reliable and non-invasive method for the preoperative prediction of HGP is urgently needed to guide individualized surgical planning.

Radiomics has emerged as a promising non-invasive imaging approach for quantitatively characterizing tumor phenotypes and has shown potential in oncologic diagnosis, risk stratification, and outcome prediction (14). Recent comprehensive reviews have highlighted the expanding role of artificial intelligence and CT radiomics, combined with clinical and multimodal imaging information, in precision oncology and individualized risk stratification for patients with lung cancer (15). Previous studies have explored CT radiomics-based models for predicting aggressive histological components and invasive characteristics in lung adenocarcinoma, suggesting that quantitative imaging biomarkers may provide valuable preoperative information beyond conventional radiological assessment (16). However, conventional radiomics primarily relies on feature extraction from the entire tumor volume of interest, which may obscure spatial variations within tumors and limit the representation of complex intratumoral heterogeneity (14).

To address this limitation, increasing attention has been directed toward imaging-based intratumoral heterogeneity (ITH) analysis. Habitat imaging and subregional clustering methods divide tumors into multiple regions with similar imaging phenotypes, enabling quantitative assessment of spatial heterogeneity within the tumor microenvironment (17). Recent studies have shown that ITH-derived quantitative metrics are associated with aggressive pathological characteristics and may provide complementary predictive information beyond conventional whole-tumor radiomics (18). Nevertheless, most existing ITH approaches focus on describing spatial variation among intratumoral regions, whereas quantitative descriptors of overall tumor structural complexity have been less frequently integrated into predictive models (16, 19).

Fractal analysis provides an additional quantitative framework for describing the structural complexity and spatial organization of complex biological structures. Unlike conventional radiomics, which primarily characterizes imaging phenotypes through predefined quantitative features, including intensity- and texture-based descriptors (14), three-dimensional (3D) fractal analysis quantifies geometric complexity across multiple spatial scales and may provide complementary information regarding whole-tumor architecture (19, 20). Previous studies have demonstrated the potential of fractal analysis as an imaging biomarker for quantitatively characterizing tumor morphology and structural complexity (19–21). Because intratumoral heterogeneity and global structural complexity reflect distinct yet complementary aspects of tumor organization, combining these approaches may enable a more comprehensive characterization of tumor imaging phenotype (18–21). Specifically, radiomic features mainly capture quantitative variations in voxel intensity and texture patterns, ITH features describe spatial differences among intratumoral regions, whereas fractal features provide a multiscale description of tumor architectural complexity and spatial organization (14, 17–20). These imaging-derived characteristics may reflect different aspects of tumor biology, including spatial heterogeneity, microenvironmental variation, and architectural complexity, which are potentially associated with the development of high-grade histological patterns.

Although radiomics, ITH analysis, and fractal features have each shown potential for tumor characterization, studies integrating these complementary imaging biomarkers with clinical information into a unified framework for preoperative HGP prediction remain limited (15–19). Clinical and conventional radiological characteristics provide macroscopic phenotypic information that complements quantitative imaging biomarkers, while radiomics, ITH, and fractal analysis capture tumor characteristics at different spatial scales. Therefore, a multimodal fusion strategy integrating these heterogeneous information sources may provide complementary information from multiple feature domains and facilitate a more comprehensive representation of tumor biology. Furthermore, the clinical application of machine learning-based prediction models may be hindered by limited interpretability, as the contribution of individual imaging biomarkers to model decisions is often unclear. In this context, explainable artificial intelligence approaches, such as SHapley Additive exPlanations (SHAP), can improve model transparency by elucidating the contribution of individual features to model predictions and facilitating clinical understanding of predictive outcomes (22). Therefore, this study aimed to develop an interpretable multimodal fusion model integrating clinical information and quantitative CT features (including radiomics, intratumoral heterogeneity, and three-dimensional fractal features) for preoperative prediction of HGP in IAC.

## 2 Materials and methods

This study was approved by the Institutional Review Boards of the Second Affiliated Hospital of Harbin Medical University (Ethics No. YJSKY2025-096) and the Fourth Hospital of Harbin (Ethics No. KY2025-07). The requirement for written informed consent was waived due to the retrospective design.

### 2.1 Patients

This retrospective study included patients with surgically resected and pathologically confirmed IAC from two centers: the Second Affiliated Hospital of Harbin Medical University (2020–2025) and the Fourth Hospital of Harbin (2022–2025). All the images and data were securely extracted from electronic records to maintain patient privacy confidentiality and ethical standards.

**Figure 1:**
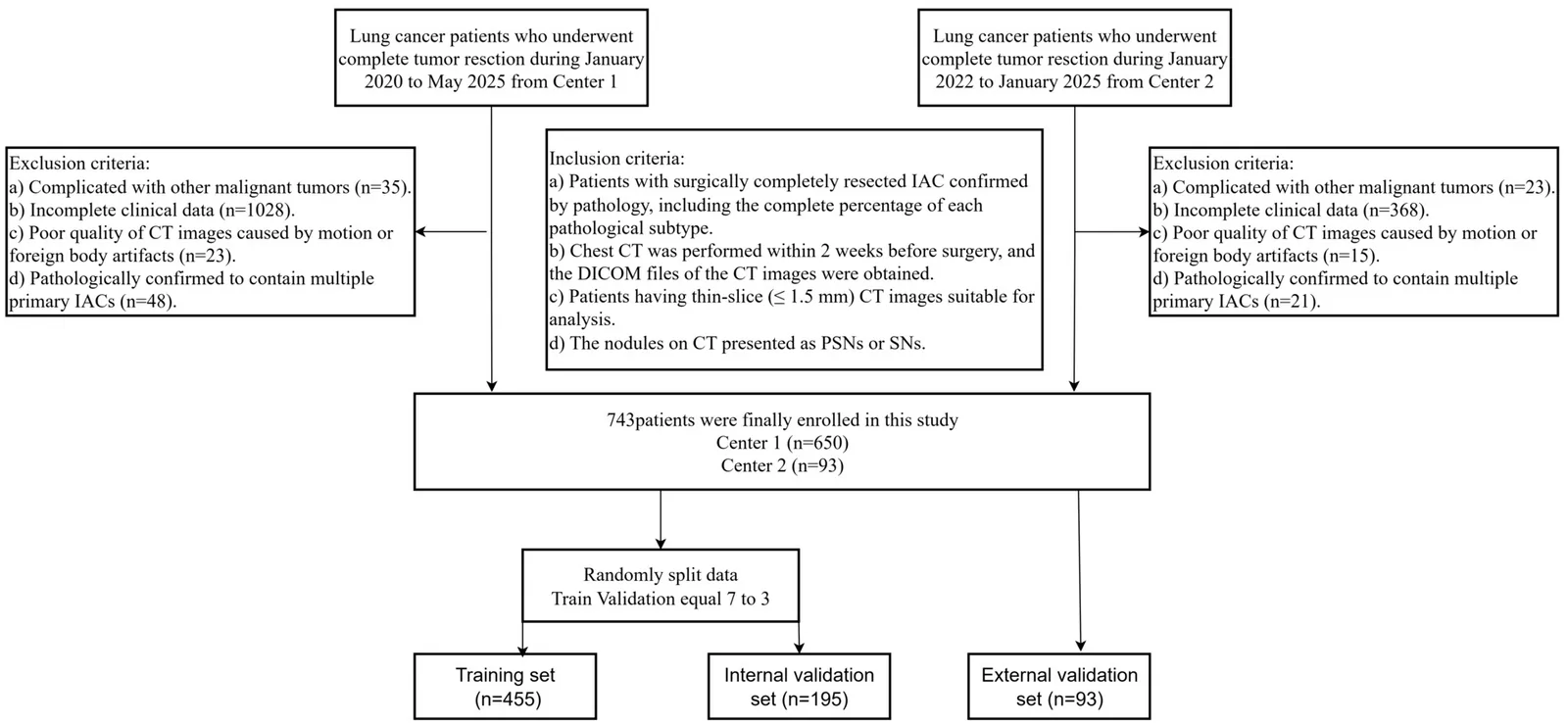
The flowchart of inclusion and exclusion criteria of patients. IAC, invasive adenocarcinoma; PSNs, part-solid nodules; SNs, solid nodules. Center 1: The Second Affiliated Hospital of Harbin Medical University; Center 2: The Fourth Hospital of Harbin.

Inclusion criteria: (1) completely resected IAC confirmed by pathology with quantitative histopathological subtype information; (2) chest CT was performed within 2 weeks before surgery with available DICOM data; (3) thin-slice (≤ 1.5 mm); (4) part-solid nodules or solid nodules. Exclusion criteria: (1) concurrent malignant tumors; (2) incomplete clinical data; (3) poor quality CT images due to motion or foreign body artifacts; (4) multiple primary IACs. A total of 743 patients were included. The Center 1 cohort was randomly assigned to a training set (n = 455) and an internal validation set (n = 195) in a 7:3 ratio, while the Center 2 cohort served as an independent external validation set (n = 93). The detailed patient selection process is illustrated in **Figure 1**.

### 2.2 Histopathological data

Surgically resected specimens were fixed in 10% formalin, embedded in paraffin, sectioned at 4 μm, and stained with hematoxylin and eosin. Two experienced pathologists independently reviewed all slides. Histologic subtypes were classified according to the 2021 World Health Organization classification of lung tumors, and each component was semiquantitatively assessed in 5% increments. Tumors were stratified into HGP (+) and HGP (-) groups based on the presence of micropapillary, solid, and complex glandular patterns.

### 2.3 Examination methods

All patients underwent CT scanning within 2 weeks prior to surgery using 256-slice or 128-slice Philips spiral CT scanner. Standardized breath-hold training was performed before examination. Scans were acquired in the supine position during a single end-inspiratory breath-hold. The scanning range extended from the lung apices to the bases, including the bilateral axillae and chest wall. Acquisition parameters were as follows: slice thickness, 1 mm; reconstruction interval, 5 mm; tube voltage, 120 kV; matrix size, 512 × 512; and bone reconstruction algorithm.

### 2.4 Radiological analysis

CT imaging features were independently evaluated by two senior radiologists based on retrospective analysis of thin-section CT images, and discrepancies were resolved by consensus. Radiological features included location, maximum diameter, density, consolidation-to-tumor ratio, lesion margin, air bronchogram sign, vacuole sign, vascular convergence sign, spiculation or lobulation sign, bronchial cutoff sign, and pleural retraction sign. The spiculation or lobulation feature was recorded as present when either spiculation or lobulation was observed. The definitions of the radiological features are summarized in **Table 1**.

**Table 1:** The definition of radiological characteristics in the study.

Radiological features	Description
Location	The distribution of each lesion in the lung was recorded, encompassing LL and RL.
Maximum diameter	Defined as the longest dimension of the lesion observed in its largest cross-sectional image.
Density	Represented by the mean Hounsfield Unit (HU) value measured at the largest cross-section of the tumor. Divided into three levels: 1 (density< -400 HU), 2 (-400 HU ≤ density< -100 HU), 3 (density ≥ -100 HU).
CTR	Calculated as the ratio of the solid component’s maximum diameter to the nodule’s maximum diameter. Divided into four levels: 1 (CTR< 25%), 2 (25% ≤ CTR< 50%), 3 (50% ≤ CTR< 90%), 4 (CTR = 100%).
Lesion margin	The margin was classified as well-defined (clearly demarcated from the surrounding lung parenchyma) or ill-defined (indistinct contour).
Air bronchogram sign	Dilated bronchial tubes pass through the interior or along the margins of the lesion.
Vacuole sign	There are hypodense areas containing air, sized 1–3 mm, within the lesion.
Vascular convergence sign	Branch vessels penetrate the nodule and then exhibit stenosis or occlusion.
Lobulation sign	The nodule’s surface appears uneven, resembling the lobes of a leaf.
Spiculation/Lobulation sign	Presence of spiculated margins characterized by linear strands extending into the surrounding lung parenchyma and/or lobulated contours characterized by one or more convex bulges along the nodule margin
Bronchial cutoff sign	A segmental, subsegmental, or more peripheral bronchial lumen, is identified no visible continuation of the bronchus beyond the point of cutoff.
Pleural retraction sign	Caused by a pulmonary nodule adhering to and pulling the adjacent pleura, resulting in a linear or curtain-shaped dense shadow.

LL, left lung; RL, right lung; HU, Hounsfield unit; CTR, consolidation-to-tumor ratio. Lower CTR values indicate a higher proportion of ground-glass opacity (GGO) components, whereas values closer to 1 indicate a more solid lesion.

### 2.5 Data processing and model construction

Tumor Processing and Segmentation: CT images were preprocessed using the OneKey AI (RRID: SCR_025655) research platform and resampled to an isotropic voxel size of 1 × 1 × 1 mm³. Tumor segmentation was performed using 3D Slicer (v5.6.2, RRID: SCR_005619). Lesions were initially localized by a radiologist using semi-automated methods and refined manually. Final contours were reviewed by a second thoracic radiologist (10 years of experience). Non-tumoral structures (vessels, bronchi, cystic air spaces, and chest wall tissue) were excluded. Interobserver reproducibility was assessed using intraclass correlation coefficients. Thirty cases were re-segmented after one month, and features with intraclass correlation coefficient > 0.75 were retained.

3D Fractal Dimension and Abundance Analysis: tumor morphological complexity was quantified using a 3D box-counting method. A cubic grid with side length s was applied to the tumor mask, and the number of occupied boxes *N*(*s*) were calculated across scales. The fractal dimension was defined as the slope of *log N*(*s*) versus *log*(1/*s*), as described in Equation 1. Fractal abundance was introduced to characterize multiscale spatial distribution. Box sizes were grouped into small (1–3 voxels), medium (4–7 voxels), and large (8–15 voxels). For each scale range, the average number of boxes containing tumor voxels was calculated and then normalized to the total number of tumor voxels.

(1) $$FD=\frac{d\log N\left(s\right)}{d\log\left(1/|s\right)}$$

Tumor Subregion Clustering Analysis and feature extraction: A combined 3D–2D framework was used to characterize intratumoral heterogeneity at complementary spatial scales. The 3D analysis was designed to capture global volumetric heterogeneity and the spatial organization of intensity-based tumor habitats across the entire segmented tumor volume, whereas the 2D analysis was used to assess local spatial heterogeneity at a higher spatial resolution within a representative tumor cross-section. For the 2D analysis, the axial slice with the largest tumor cross-sectional area was selected as a representative plane containing the maximal tumor extent, thereby enabling detailed pixel-level analysis using a sliding-window approach. This approach also reduced the computational complexity and potential variability associated with applying local sliding-window analysis across the entire tumor volume. Thus, the 3D and 2D components were considered complementary rather than redundant. For the 3D analysis, voxel-wise intensity distributions within each segmented tumor volume were first normalized to reduce inter-patient intensity variation. Each voxel was represented by its normalized intensity value, and hierarchical density-based spatial clustering of applications with noise (HDBSCAN) was applied to identify intensity-based tumor habitats. Four predefined combinations of HDBSCAN parameters (minimum cluster size and minimum samples) were evaluated, and the optimal clustering result was selected according to the highest silhouette score. Voxels identified as noise by HDBSCAN were excluded from subsequent habitat characterization. The remaining clusters were regarded as distinct intratumoral habitats, from which quantitative descriptors, including the number of habitats, cluster volume ratio, Davies–Bouldin index, and Calinski–Harabasz index, were calculated. For complementary 2D analysis, the largest cross-sectional tumor slice was selected for pixel-wise clustering. Local radiomic features were extracted from the largest tumor cross-section using a sliding-window strategy, and pixel-wise clustering was subsequently performed based on these local radiomic feature vectors for cluster numbers (K) ranging from 2 to 6. For each clustering configuration, the resulting label map was used to calculate an ITH score using the ITHscore package, yielding ITH_Score_K2 to ITH_Score_K6 as quantitative measures of intratumoral spatial heterogeneity. Radiomic features were extracted using PyRadiomics (RRID: SCR_026019) in accordance with the Image Biomarker Standardisation Initiative guidelines. Both original and filtered images (Laplacian of Gaussian and wavelet) were included. Features comprised first-order statistics, shape descriptors, and texture features derived from GLCM, GLRLM, GLSZM, GLDM, and NGTDM. A total of 1, 684 radiomic features were extracted for each tumor. Habitat-derived descriptors and ITH scores were subsequently combined with radiomic and clinical variables for downstream feature selection and model construction.

Feature Processing and Model Construction: feature preprocessing was performed using the training cohort only to prevent information leakage. missing rates were calculated in the training cohort, and features with missing rate > 25% were excluded. Missing values in the external validation cohort were imputed using training-derived strategies. Continuous variables were standardized using Z-score normalization, and categorical variables were encoded using one-hot encoding. A two-stage feature selection strategy was applied: In the first stage, eight supervised feature selection methods were evaluated independently, including minimum redundancy maximum relevance, analysis of variance F-test, Kendall correlation, Pearson correlation, SelectPercentile, Spearman correlation, variance thresholding, and recursive feature elimination. In the second stage, Least Absolute Shrinkage and Selection Operator regression with L1 regularization was applied to further eliminate redundant variables. Ten machine learning classifiers were subsequently evaluated, including Logistic Regression, Linear Support Vector Machine, Radial Basis Function Support Vector Machine, Random Forest, Extra Trees, Extreme Gradient Boosting, Gradient Boosting Machine, Multilayer Perceptron, Gaussian Naïve Bayes, and k-Nearest Neighbors. Combining the eight first-stage feature selection methods with the ten classifiers generated 80 candidate models. For classifiers supporting class weighting, class imbalance was addressed by assigning balanced class weights during model training. Hyperparameters for both feature selection and classifiers were optimized using grid search with five-fold stratified cross-validation performed exclusively within the training cohort. Feature selection, preprocessing, hyperparameter optimization, and classifier training were integrated into a single scikit-learn pipeline, ensuring that all preprocessing and feature selection procedures were fitted independently within each cross-validation training fold before being applied to the corresponding validation fold, thereby minimizing information leakage. No information from the internal or external validation cohorts was used during preprocessing, feature selection, hyperparameter optimization, or model training. Among the 80 candidate models, the final model was selected based primarily on the highest cross-validation area under the receiver operating characteristic curve (AUC), with calibration performance and external validation performance considered comprehensively.

Model Evaluation and Interpretation: the final selected model was evaluated in both the internal and external validation cohorts. Discrimination was assessed using AUC with 95% confidence intervals. Pairwise comparisons of AUCs among different models were performed using the DeLong test. Sensitivity, specificity, accuracy, positive predictive value, and negative predictive value were calculated at the optimal threshold determined by the Youden index. Calibration was assessed using calibration curves and the Hosmer–Lemeshow test. Clinical utility was evaluated using decision curve analysis. Given the potential application of preoperative HGP risk prediction for individualized risk stratification and surgical or management decision-making, the decision curve analysis results were interpreted with particular attention to a clinically plausible threshold probability range of 20%–70%, while the full threshold probability range from 0% to 100% was retained for completeness. Model interpretability was assessed using SHAP, and values for one-hot encoded variables were aggregated at the original feature level.

### 2.6 Statistical analysis

The normality of all continuous variables was assessed using the Shapiro–Wilk test. Non-normally distributed continuous variables, such as age and tumor size, were presented as the median and interquartile range, and intergroup differences among the training, internal validation, and external test cohorts were evaluated using the Kruskal–Wallis H test. Categorical variables, including clinical characteristics, tumor stage, and outcome labels, were summarized as frequencies and percentages. Pearson’s chi-square test was used to compare the distribution of categorical variables across cohorts. When the expected frequency in any cell was less than 5 (e.g., for the CA15–3 variable with a very small sample size), Fisher’s exact test was applied. A two-sided p value< 0.05 was considered statistically significant. All data preprocessing and statistical analyses were conducted using Python (version 3.8, RRID: SCR_026019).

## 3 Results

### 3.1 Patient characteristics

A total of 650 patients from Center 1 were randomly divided into a training cohort (n = 455) and an internal validation cohort (n = 195). An independent external cohort of 93 patients was included from Center 2. The distribution of HGP (+) and HGP (-) cases was comparable among the training, internal validation, and external validation cohorts (chi-square test, p = 0.998), indicating that the cohort split preserved the outcome distribution. The median age was similar across cohorts, and no significant differences were observed in clinical characteristics between HGP (+) and HGP (−) groups (all p > 0.05). Clinical characteristics and radiological features and are summarized in **Tables 2**, **3**.

**Table 2:** Clinical characteristics of training, validation, and test cohort at baseline.

Clinical characteristics	Train-cohort (n=455)	Validation cohort (n=195)	Test cohort (n=93)	p-value
Gender				0.417^†^
Female	310 (68.1)	125 (64.1)	58 (62.4)	
Male	145 (31.9)	70 (35.9)	35 (37.6)	
Age (years)*	62.00 [56.00-68.00]	63.00 [56.00-68.00]	62.00 [57.00-67.00]	0.944^§^
Smoking				0.877^†^
Never	384 (84.4)	162 (83.1)	77 (82.8)	
Current or former	71 (15.6)	33 (16.9)	16 (17.2)	
CEA				0.101^†^
Normal	338 (74.3)	160 (82.1)	71 (76.3)	
Elevated	117 (25.7)	35 (17.9)	22 (23.7)	
CA_125				0.105^‡^
Normal	438 (96.3)	191 (97.9)	93 (100.0)	
Elevated	17 (3.7)	4 (2.1)	0 (0.0%)	
CA15_3				0.038^‡^
Normal	443 (97.4)	194 (99.5)	88 (94.6)	
Elevated	12 (2.6)	1 (0.5)	5 (5.4)	
CA19_9				0.427^†^
Normal	427 (93.8)	178 (91.3)	85 (91.4)	
Elevated	28 (6.2)	17 (8.7)	8 (8.6)	
N stage				0.806^‡^
0	418 (91.9)	182 (93.3)	83 (89.2)	
1	20 (4.4)	7 (3.6)	7 (7.5)	
2	15 (3.3)	5 (2.6)	3 (3.2)	
3	2 (0.4)	1 (0.5)	0 (0.0)	
HGP status				0.998^†^
HGP(+)	225 (49.5%)	97 (49.7%)	46 (49.5%)	
HGP(-)	230 (50.5%)	98 (50.3%)	47 (50.5%)	

Except where indicated, data are numbers of patients, with percentages in parentheses. CEA, carcinoembryonic antigen; NSE, neuron-specific enolase; N stage, Clinical N stage. P-value< 0.05 indicates a statistically significant difference. * Data are medians, with IQRs in parentheses; ^†^Chi-square test; ^‡^Fisher’s exact test; ^§^Kruskal–Wallis test.

**Table 3:** Radiological characteristics of training, validation, and test cohort at baseline.

Radiological characteristics	Train-cohort (n=455)	Validation cohort (n=195)	Test cohort (n=93)	P-value
Location				0.126^†^
RL	269 (59.1%)	110 (56.4%)	64 (68.8%)	
LL	186 (40.9%)	85 (43.6%)	29 (31.2%)	
Max diameter*	19.00 [14.00-25.00]	18.00 [13.00-24.50]	19.00[14.00-26.00]	0.345^§^
Density				0.435^†^
low density	61 (13.4)	31 (15.9)	15 (16.1)	
medium density	153 (33.6)	70 (35.9)	38 (40.9)	
high density	241 (53.0)	94 (48.2)	40 (43.0)	
CTR				0.74^†^
low	77 (16.9)	35 (17.9)	17 (18.3)	
sub-low	56 (12.3)	29 (14.9)	16 (17.2)	
sub-high	229 (50.3)	98 (50.3)	46 (49.5)	
high	93 (20.4)	33 (16.9)	14 (15.1)	
boundaries				0.476^†^
No	357 (78.5)	161 (82.6)	73 (78.5)	
Yes	98 (21.5)	34 (17.4)	20 (21.5)	
Air bronchogram sign				0.823^†^
No	236 (51.9)	96 (49.2)	47 (50.5)	
Yes	219 (48.1)	99 (50.8)	46 (49.5)	
Vacuole sign				0.675^†^
No	283 (62.2)	127 (65.1)	56 (60.2)	
Yes	172 (37.8)	68 (34.9)	37 (39.8)	
Vascular convergence sign				0.724^†^
No	66 (14.5)	24 (12.3)	14 (15.1)	
Yes	389 (85.5)	171 (87.7)	79 (84.9)	
Spiculation/Lobulation sign				0.631^†^
No	36 (7.9)	18 (9.2)	10 (10.8)	
Yes	419 (92.1)	177 (90.8)	83 (89.2)	
bronchial cut off sign				0.122^†^
No	89 (19.6)	47 (24.1)	13 (14.0)	
Yes	366 (80.4)	148 (75.9)	80 (86.0)	
Pleural retraction sign				0.059^†^
No	117 (25.7)	64 (32.8)	33 (35.5)	
Yes	338 (74.3)	131 (67.2)	60 (64.5)	

Data are presented as mean ± standard deviation or n(%). P-value< 0.05 indicates a statistically significant difference. * Data are medians, with IQRs in parentheses; ^†^Chi-square test; ^‡^Fisher’s exact test; ^§^Kruskal–Wallis test.

### 3.2 Model performance evaluation

**Figure 2:**
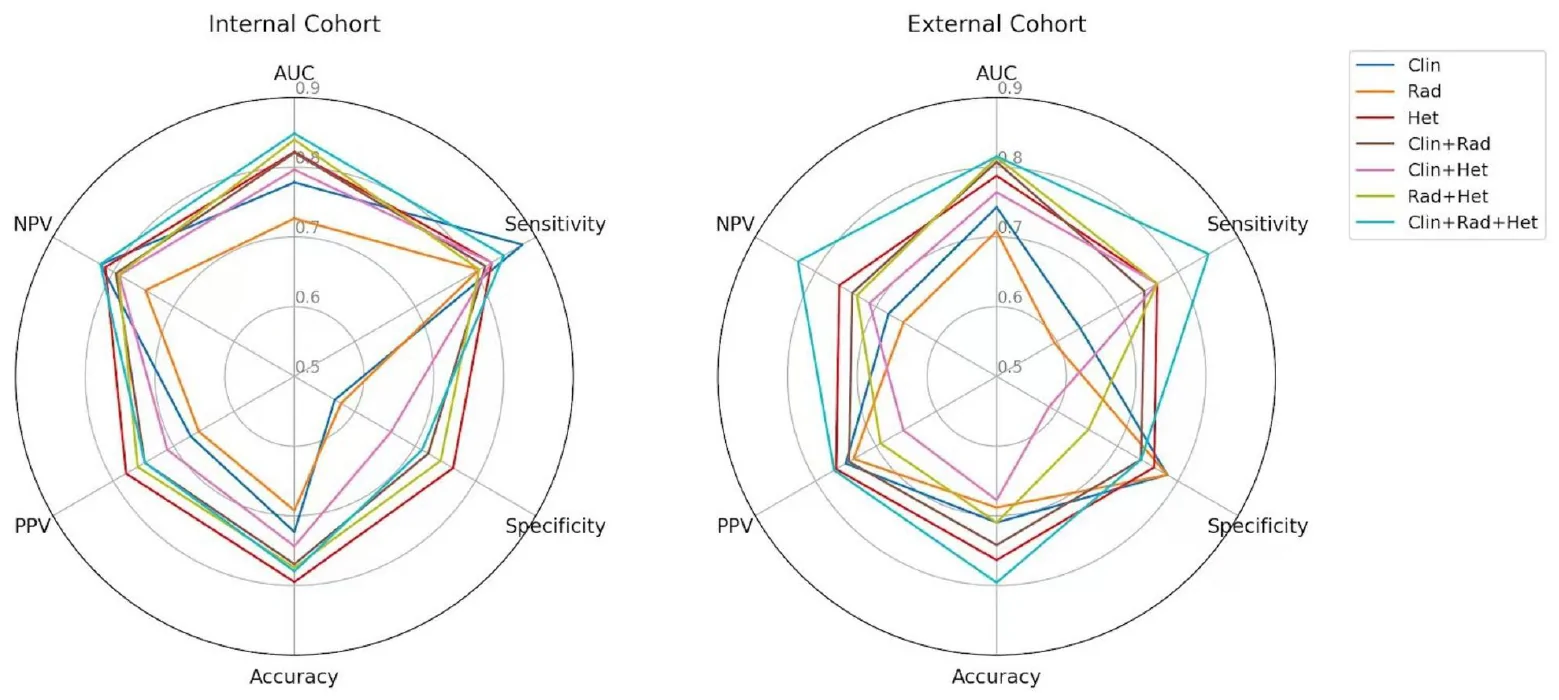
Radar plot of the clinical, radiomics, heterogeneity, and combined models in the internal and external validation cohorts. Clin, clinical model; Rad, radiomics model; Het, heterogeneity model.

**Figure 3:**
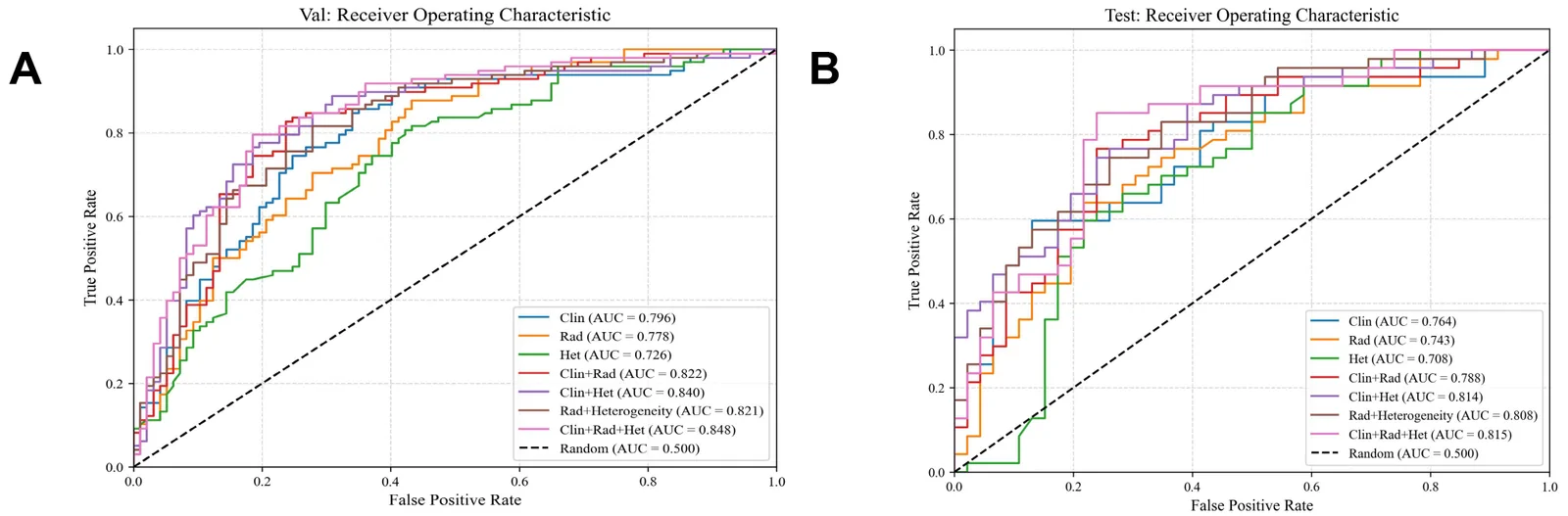
ROC curves **(A, B)** of the clinical, radiomics, heterogeneity, and combined models in the internal and external validation cohorts. Clin, clinical model; Rad, radiomics model; Het, heterogeneity model.

**Figure 4:**
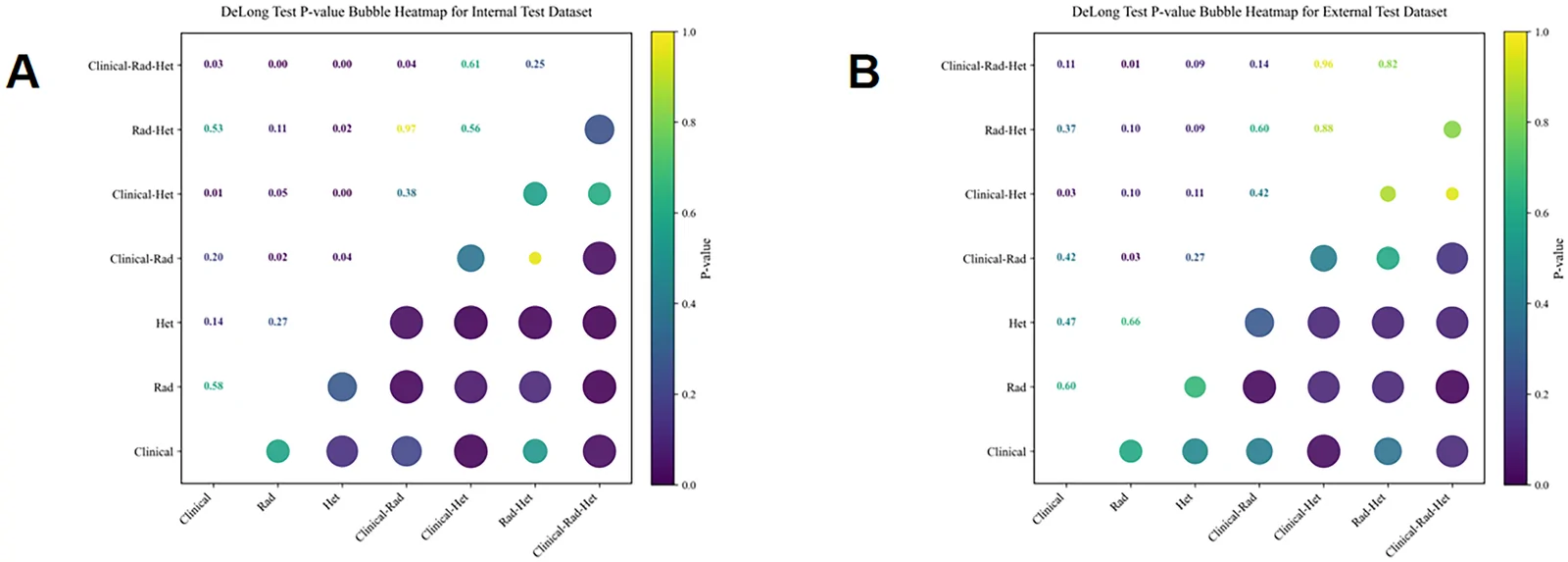
DeLong tests **(A, B)** of the clinical, radiomics, heterogeneity, and combined models in the internal and external validation cohorts. The numbers indicate the P values for pairwise comparisons of the AUCs. Bubble color and size correspond to the P values. Clin, clinical model; Rad, radiomics model; Het, heterogeneity model.

**Figure 5:**
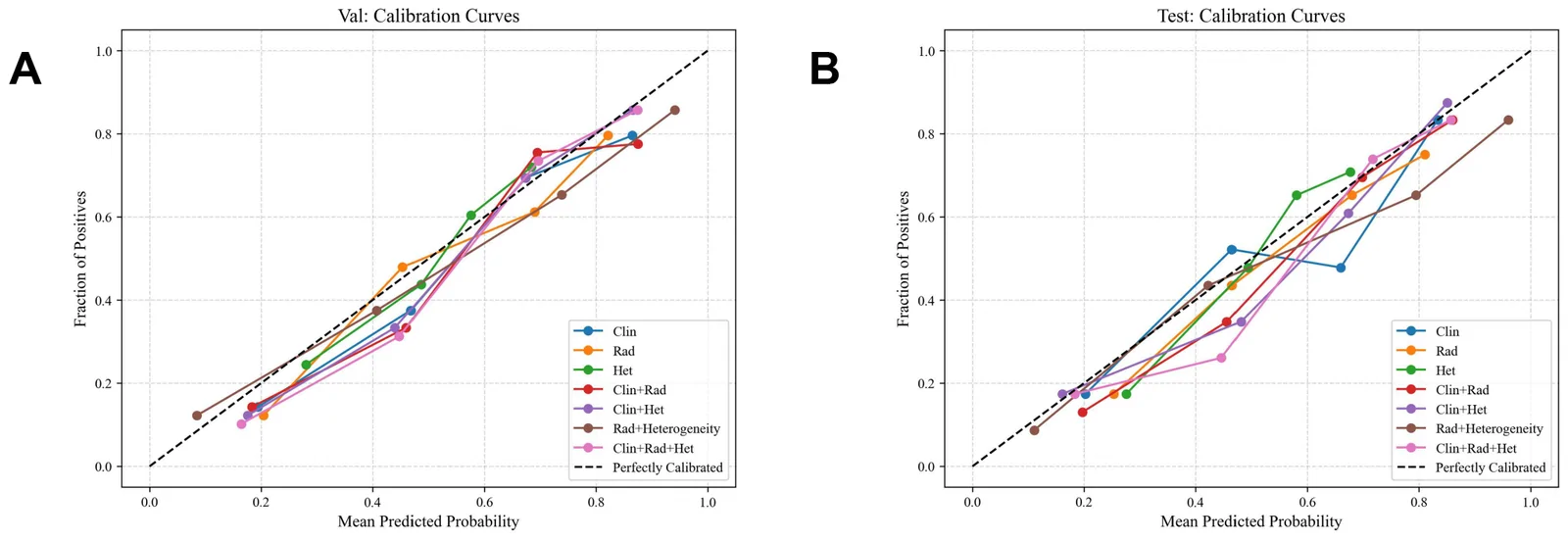
Calibration curves **(A, B)** of the clinical, radiomics, heterogeneity, and combined models in the internal and external validation cohorts. Clin, clinical model; Rad, radiomics model; Het, heterogeneity model.

**Figure 6:**
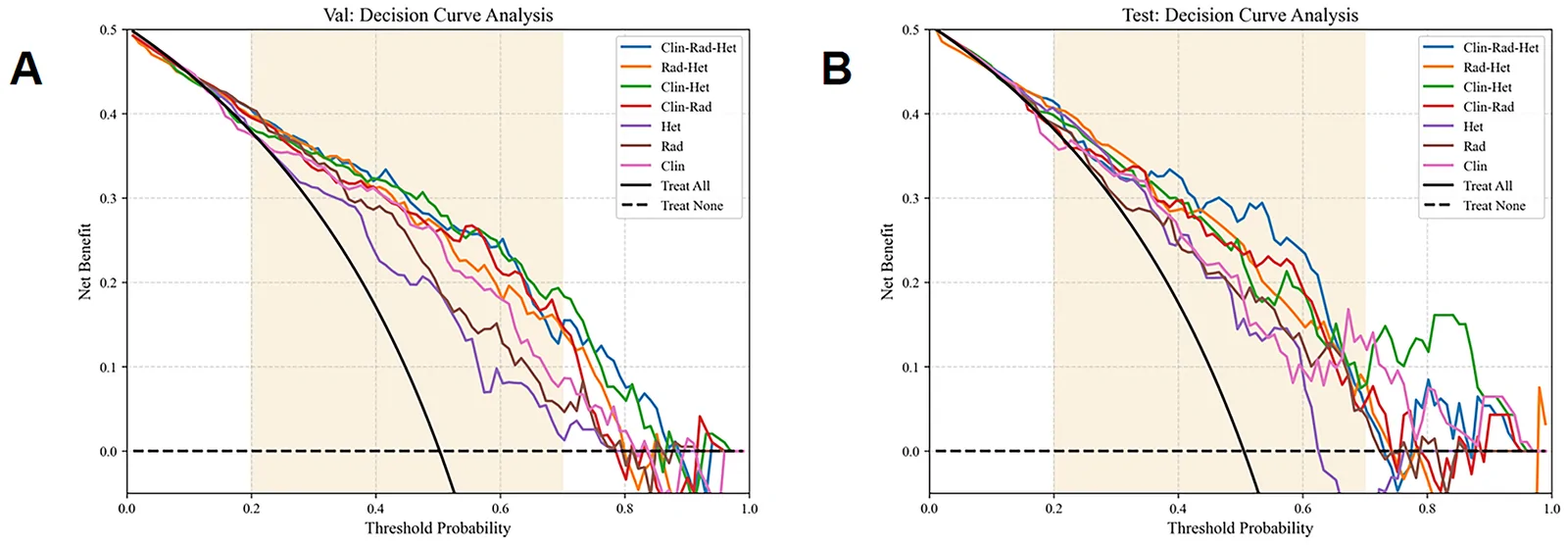
DCA **(A, B)** of the clinical, radiomics, heterogeneity, and combined models in the internal and external validation cohorts. Clin, clinical model; Rad, radiomics model; Het, heterogeneity model.

Based on the comprehensive evaluation using ROC curves, calibration curves, decision curve analysis, and radar plot analysis (**Figure 2**), the combined model (Clin+Rad+Het) achieved the best performance in both the internal and external cohorts. In the internal validation cohort, the model achieved an AUC of 0.848 (**Figure 3A**), outperforming Clin+Rad (0.822), Clin+Het (0.840), and Rad+Het (0.821) models (**Table 4**). In the external test cohort, it achieved an AUC of 0.815 (**Figure 3B**), compared with AUCs of 0.788, 0.814, and 0.808 for the Clin+Rad, Clin+Het, and Rad+Het models, respectively (**Table 5**). DeLong tests were performed to compare the discriminative performance of different models. In the internal validation cohort, the Clin+Rad+Het model significantly outperformed the Clin+Rad model (P = 0.04), indicating that incorporation of the heterogeneity module (ITH scores and fractal features) provided significant incremental predictive value beyond conventional clinical-radiomics features. No significant differences were observed between the Clin+Rad+Het model and the Clin+Het (P = 0.61) or Rad+Het (P = 0.25) models (**Figure 4A**). In the external validation cohort, although the Clin+Rad+Het model achieved the highest AUC (0.815), its performance was not significantly different from that of the Clin+Rad model (P = 0.14), which may be attributable to the limited sample size of the external cohort and the resulting reduced statistical power (**Figure 4B**). Calibration curves demonstrated excellent agreement between the predicted and observed outcomes in the external test cohort, with the scatter points closely aligned with the ideal calibration line (**Figures 5A, B**). Decision curve analysis demonstrated that the combined Clin+Rad+Het model provided a favorable net clinical benefit across a substantial portion of the clinically plausible threshold probability range of 20%–70% in both the internal and external validation cohorts (**Figures 6A, B**). Although the relative net benefit varied across individual threshold probabilities and some model curves overlapped or crossed, particularly between the combined model and the Clin+Het model, the combined model generally demonstrated a favorable overall net benefit compared with most alternative models.

**Table 4:** Performance of the predictive model in the validation cohort.

Validation cohort	AUC(95% CI)	Sensitivity	Specificity	Accuracy	PPV	NPV
Clin	0.796 (0.734-0.853)	0.827	0.660	0.744	0.711	0.790
Rad	0.778 (0.708-0.845)	0.878	0.567	0.723	0.672	0.821
Het	0.726 (0.660-0.804)	0.806	0.577	0.692	0.658	0.747
Clin+Rad	0.822 (0.763-0.884)	0.827	0.763	0.795	0.779	0.813
Clin+Het	0.840 (0.784-0.896)	0.806	0.742	0.774	0.760	0.791
Rad+Het	0.821 (0.757-0.881)	0.816	0.722	0.769	0.748	0.795
Clin+Rad+Het	0.848 (0.798-0.906)	0.847	0.711	0.779	0.748	0.821

Clin, clinical model; Rad, radiomics model; Het, heterogeneity model; AUC, area under the receiver operating characteristic curve; CI, confidence interval; PPV, positive predictive value; NPV, negative predictive value.

**Table 5:** Performance of the predictive model in the test cohort.

Test cohort	AUC(95% CI)	Sensitivity	Specificity	Accuracy	PPV	NPV
Clin	0.764 (0.656-0.850)	0.766	0.587	0.677	0.655	0.711
Rad	0.743 (0.624-0.840)	0.638	0.783	0.710	0.750	0.679
Het	0.708 (0.607-0.813)	0.596	0.783	0.688	0.737	0.655
Clin+Rad	0.788 (0.687-0.879)	0.766	0.761	0.763	0.766	0.761
Clin+Het	0.814 (0.725-0.892)	0.766	0.652	0.710	0.692	0.732
Rad+Het	0.808 (0.717-0.892)	0.745	0.739	0.742	0.745	0.739
Clin+Rad+Het	0.815 (0.720-0.898)	0.851	0.739	0.796	0.769	0.829

Clin, clinical model; Rad, radiomics model; Het, heterogeneity model; AUC, area under the receiver operating characteristic curve; CI, confidence interval; PPV, positive predictive value; NPV, negative predictive value.

**Figure 7:**
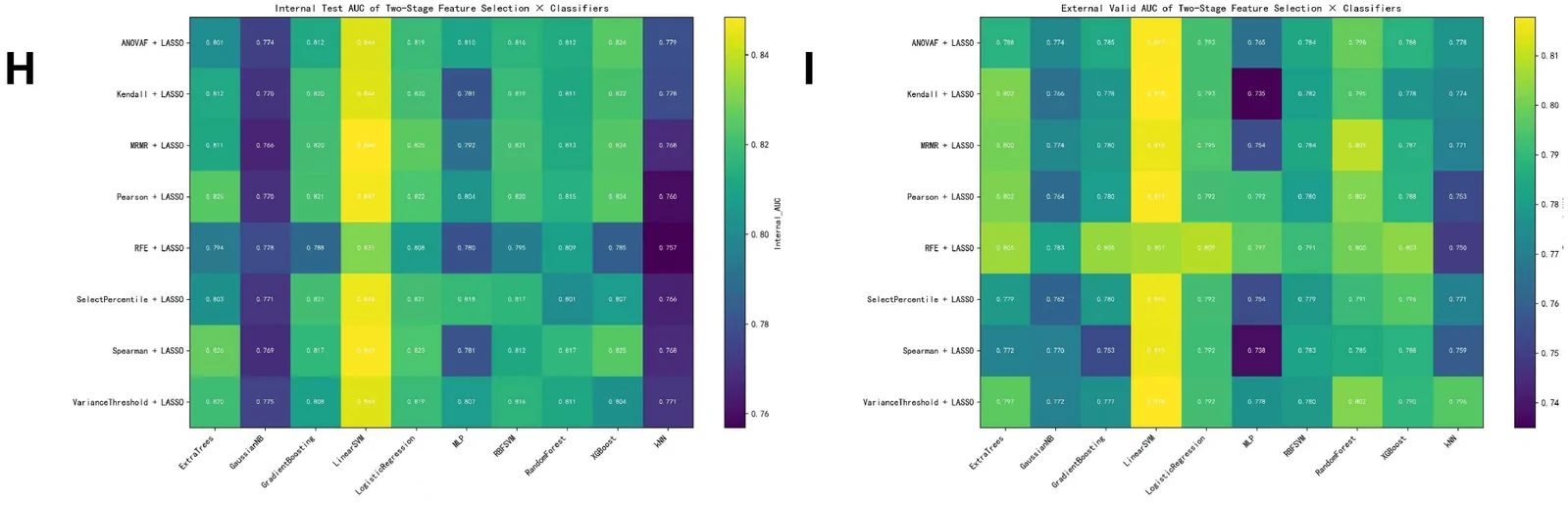
Heatmap **(A, B)** of the clinical, radiomics, heterogeneity, and combined models in the internal and external validation cohorts. Clin, clinical model; Rad, radiomics model; Het, heterogeneity model.

The superior performance of the combined model is attributed to the global screening of 80 algorithm combinations (**Figures 7A, B**). Heatmap analysis indicated that the classifier selection had an important influence in model performance, with most conventional combinations achieving AUCs of 0.74-0.79. The best performance was achieved using a linear support vector machine with maximum relevance minimum redundancy feature selection followed by least absolute shrinkage and selection operator, yielding AUCs of 0.848 and 0.815 in the internal and external validation cohorts, respectively.

### 3.3 Model interpretation

**Figure 8:**
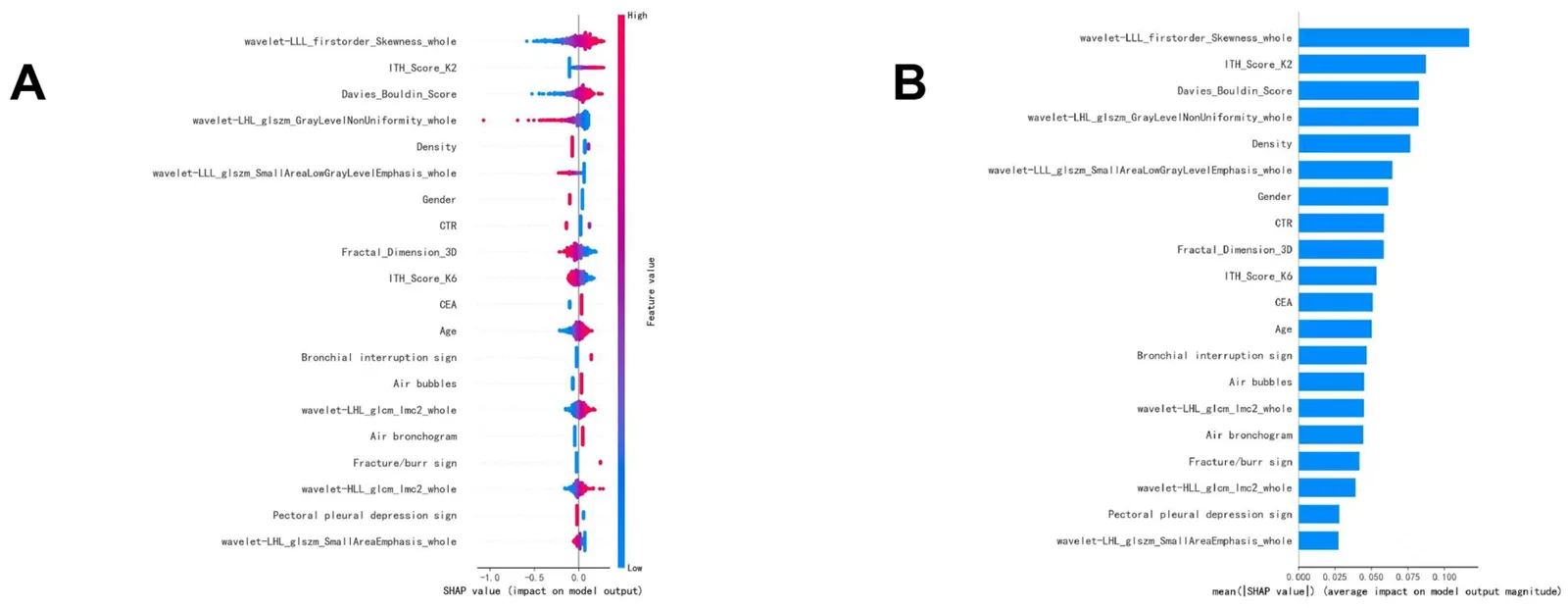
Model interpretability is visualized through SHAP analysis. **(A)** (SHAP summary plot) illustrates the overall importance of the features across the model and clearly illustrates their directional impact (red indicates high feature values, blue indicates low feature values, and the horizontal axis represents the magnitude of each feature’s contribution to the model output). **(B)** (SHAP bar plot) presents the top 20 most important features, with the horizontal axis representing the mean absolute SHAP values, thereby providing a clear ranking of feature importance in terms of their contribution to the overall model prediction.

The SHAP summary plot demonstrated the global contribution and directional effects of each feature on model predictions (**Figures 8A, B**). Feature importance analysis indicated that multiscale wavelet features, first-order statistical features, and tumor heterogeneity and clustering metric contributed most to the model. Wavelet-LLL_firstorder_Skewness_whole showed the highest contribution, with higher values associated with increased model output. ITH-related features and the Davies–Bouldin index showed positive associations with model output, whereas 3D fractal Dimension showed a negative association. Clinical and radiological features had limited contributions. Overall, fractal- and habitat-based features were important contributors within the heterogeneity module and may provide complementary information regarding tumor spatial heterogeneity and architectural complexity.

## 4 Discussion

Studies have shown that the presence of HGP is an independent risk factor for recurrence and poor prognosis (23). Furthermore, the survival outcomes for these patients are significantly influenced by the surgical resection strategy. Although the latest IASLC grading system defines poorly differentiated invasive LUAD as HGP ≥ 20%, several studies indicate that even a low proportion of micropapillary or solid patterns components (≤ 5%) is associated with higher rates of lymph node metastasis and visceral pleural invasion, as well as significantly reduced overall survival (7, 24, 25). Clinically, micropapillary or solid patterns (+) patients frequently present with higher SUVmax values and larger tumor volumes, and an increased risk of tumor spread through air spaces, all of which are associated with more aggressive biological behavior (26, 27). Therefore, the preoperative prediction of HGP holds significant clinical importance for formulating individualized treatment strategies and follow-up management in patients with IAC.

Although previous studies have demonstrated the prognostic value of radiomics features in predicting HGP in patients with LUAD, existing models still have several limitations. For instance, Dong et al. developed a radiological-radiomics model using multivariable logistic regression, achieving AUCs of 0.923 and 0.920 in the training and validation cohorts, respectively, but the model relied solely on a single logistic regression algorithm (3). He et al. constructed and compared machine learning models for predicting micropapillary or solid patterns components, but the external validation performance remained relatively modest, with AUCs ranging from 0.69 to 0.72 (23). Recently, subregion-based Het quantification methods have demonstrated considerable promise. Zuo et al. constructed a hybrid model incorporating ITH scores extracted from tumor subregions and achieved an AUC of 0.849 in the test cohort (18), suggesting that subregional analysis may better capture the spatial heterogeneity of tumors compared with conventional whole-tumor radiomics.

To address the limitations of existing models in characterizing multidimensional heterogeneity, this study employed multiple machine learning algorithms. Building upon clinical and radiological features, we developed a Het module by combining three-dimensional fractal descriptors with ITH scores derived from tumor subregion clustering, enabling complementary characterization of both global structural complexity and regional spatial heterogeneity. Fractal dimension analysis quantifies the self-similarity, geometric complexity, and space-filling capacity of tumor boundaries and internal architectures, thereby capturing structural heterogeneity that may not be fully characterized by conventional texture features (28, 29). Meanwhile, subregion clustering analysis delineates tumor habitats from the perspective of functional heterogeneity (30). The multiscale fusion of these approaches not only characterizes variations in spatial distribution but also provides a more comprehensive representation of tumor heterogeneity across multiple spatial scales. Our results demonstrated that the proposed combined model achieved AUCs of 0.848 and 0.815 in the validation and external test cohorts, respectively. DeLong analysis further demonstrated that incorporating the Het module significantly improved discrimination compared with the Clin+Rad model in the internal validation cohort, supporting the incremental value of heterogeneity-related features. Moreover, the combined model demonstrated better predictive performance than the standalone clinical and conventional radiomics models. Decision curve analysis further suggested that the combined model provided favorable potential clinical utility within the clinically plausible threshold probability range of 20%–70%, generally yielding a higher net benefit than the treat-all and treat-none strategies. Although the combined model generally showed a higher net benefit than the other candidate models, the differences between models were relatively modest across some threshold ranges. Therefore, the clinical advantage of the combined model should be interpreted as an overall improvement in potential decision-support value rather than a uniformly superior net benefit at every threshold probability. Because the Het module was intentionally designed as an integrated representation combining intratumoral heterogeneity scores and three-dimensional fractal descriptors, the present study focused on evaluating the overall contribution of the heterogeneity module rather than the independent predictive value of fractal features. Therefore, the observed performance improvement should be interpreted as the incremental value of the integrated heterogeneity module instead of isolated fractal descriptors. Future studies incorporating dedicated ablation analyses are warranted to further clarify the independent contribution and incremental value of fractal descriptors beyond conventional radiomics and ITH-derived features. Compared with previous radiomics-based models, our framework extends conventional imaging analysis by integrating clinical characteristics, conventional radiomics, and multiscale heterogeneity descriptors into a unified framework. This multimodal strategy provides a more comprehensive characterization of tumor biology than approaches relying primarily on conventional radiomics or subregional heterogeneity alone. These findings suggest that combining fractal-based geometric characterization with subregion-based heterogeneity analysis may facilitate the prediction of HGP in LUAD.

The SHAP analysis demonstrated that several high-dimensional quantitative features contributed substantially to the model predictions of HGP, whereas conventional morphological features showed relatively limited contributions within the final model. Specifically, higher-order texture features (wavelet-LHL_firstorder_Skewness_whole) and subregional heterogeneity scores (ITH Score K2 and K6) made substantial positive contributions to the model output, indicating their relevance to the prediction of HGP. The Davies–Bouldin index also contributed to model predictions, reflecting differences in cluster structure and separation among tumor subregions. Among clinical variables, serum CEA levels and gender showed moderate contributions, consistent with previous studies (23). Notably, the consolidation tumor ratio showed a negative contribution in the SHAP analysis, which differed from previous findings reporting its predictive value in part-solid nodules (31, 32). This discrepancy may be attributed to the inclusion of a mixed cohort comprising both solid and part-solid nodules in the present study. Given the differences in imaging structural composition across nodule subtypes, the predictive value of consolidation tumor ratio may vary among lesion categories, which may lead to different directions of feature contribution within a mixed-sample modeling framework. Furthermore, within the multiscale fusion model, features such as the 3D fractal dimension and subregional heterogeneity scores contributed to model predictions and may reflect complementary aspects of intratumoral architectural complexity and spatial heterogeneity. Overall, these findings suggest that high-dimensional radiomic and heterogeneity-based features provide complementary information to conventional imaging features in modeling HGP. Moreover, SHAP-based interpretation enhanced the transparency of the proposed multimodal model by providing intuitive explanations of feature contributions, thereby facilitating clinical interpretation and supporting its potential application in clinical decision-making.

A major strength of this study is the systematic comparison of multiple machine learning algorithms, resulting in the selection of an optimized fusion model that demonstrated stable predictive performance across both the internal and external validation cohorts. Furthermore, SHAP analysis, an explainable artificial intelligence method based on Shapley values, quantified feature contributions and improved model interpretability by revealing both global and local feature importance (22).

The proposed multimodal fusion strategy was designed to integrate complementary information from clinical characteristics, conventional radiomics, and heterogeneity descriptors to provide a more comprehensive representation of tumor biology. In the present study, the combined model achieved the highest overall predictive performance across the evaluated models. Rather than focusing solely on maximizing predictive performance, this framework integrates complementary imaging information that captures different aspects of tumor biology. Clinical variables reflect patient and morphological characteristics, radiomics quantify global imaging phenotypes, whereas heterogeneity descriptors characterize spatial complexity within tumors. The integration of these complementary feature domains may improve the biological interpretability and robustness of the model, even when the incremental improvement in AUC is modest. The combined model showed potential clinical utility across clinically relevant threshold probabilities, although its advantage over some competing models was limited in certain decision intervals. Because clinically established decision thresholds for applying preoperative HGP prediction in surgical decision-making remain unavailable, further prospective multicenter studies are warranted to determine clinically relevant decision thresholds and to validate whether the increased model complexity translates into meaningful improvements in clinical decision-making beyond simpler models.

Several limitations should be acknowledged. Firstly, the retrospective design and specific inclusion criteria may have introduced selection bias, thereby limiting the generalizability of the findings. Secondly, although an independent external validation cohort was included, its relatively small sample size limited our ability to fully evaluate the robustness and generalizability of the proposed model. Thirdly, the peritumoral region was not included in the present analysis. Future studies should evaluate different peritumoral margins to optimize model performance. Finally, interobserver variability among pathologists in histological subtype assessment may affect quantification, particularly in tumors with minor HGP components.

## 5 Conclusion

In conclusion, the present study showed that a combined model integrating clinicoradiological features, ITH scores, and 3D fractal features, based on a linear support vector machine, may be used to predict HGP status in patients with primary IAC. This model may assist clinical decision-making and support the development of more precise and individualized therapeutic strategies.

## Data Availability

The raw data supporting the conclusions of this article will be made available by the authors, without undue reservation.
